# Diagnostic Evaluation and Cervical Spine Surgery in the Setting of a Cardiac Left Ventricular Assist Device: Challenges and a Case Illustration

**DOI:** 10.7759/cureus.19571

**Published:** 2021-11-14

**Authors:** Austin H Carroll, Ehsan Dowlati, Charles Miller, Daniel R Felbaum

**Affiliations:** 1 Department of Neurological Surgery, Georgetown University School of Medicine, Washington DC, USA; 2 Department of Neurological Surgery, MedStar Georgetown University, Washington DC, USA; 3 Department of Neurological Surgery, Walter Reed National Military Medical Center, Bethesda, USA; 4 Department of Neurosurgery, MedStar Georgetown University, Washington DC, USA

**Keywords:** orthopaedic surgery, spine surgery, cervical spine, myelogram, neurosurgery, left ventricular assist device, diagnosis

## Abstract

Due to incompatibility with magnetic resonance imaging, patients with left ventricular assist devices (LVADs) presenting with pathologies of the spinal soft tissues or neural elements represent diagnostically complex cases. We present a case of a patient undergoing a CT (computed tomography) myelogram and subsequent successful cervical posterior laminectomy. A C1-C2 lateral puncture approach CT myelogram revealed nearly a complete block of contrast movement at the level of the C2-C3 vertebrae concerning a compressive etiology. The cervical lateral approach was chosen based on patient symptomology and concern that contrast dye injected in the lumbar spine would not travel to the region of interest due to altered CSF pulsatility caused by the LVAD device. A C3-C7 posterior laminectomy was then successfully performed. Intra-operatively, however, there was no sign of a compressive lesion, and ultrasound confirmed a decompressed spinal cord. This case highlights the diagnostic challenges of pre-operative evaluation in patients with LVADs in which the efficacy of performing CT myelograms is also questionable due to potential alterations in cerebrospinal fluid movement due to variations in arterial pulsatility due to LVAD physiology.

## Introduction

Due to recent technological advances, left ventricular assist devices (LVADs) are being increasingly used as a bridge to transplant or final destination therapy in patients with advanced heart failure [1]. These patients experience unique challenges when they subsequently need non-cardiac surgical procedures or diagnostic imaging as they require constant anticoagulation and strict intra-operative LVAD parameters. They also have altered cardiovascular physiology that often requires a cardiac anesthesia specialist. Additionally, patients with the HeartMate II, HeartMate 3, and HeartWare system LVADs are unable to undergo magnetic resonance imaging (MRI) studies given the incompatibility due to the highly ferromagnetic components [2].

This becomes especially problematic in those with pathologies affecting the spine or spinal cord as imaging traditionally relies on MRI to evaluate the neural elements (spinal cord and nerve roots), ligamentous structures, and compressive etiologies such as tumors, abscesses, or degenerative spine disease. In those unable to undergo MRI, computed tomography (CT) myelography can be used to enhance visualization of the neural elements by contrasting the subarachnoid space and outlining the spinal cord and roots. Contrast is injected into the subarachnoid space typically via a lumbar puncture, and over time, the contrast diffuses throughout the subarachnoid space. A cervical puncture at the C1-C2 level can also be done when a lumbar puncture is contraindicated, the level of pathology in question is more cranially located, or it is deemed necessary to identify the superior border of the pathology.

Recently, it has been postulated that the altered circulatory physiology in LVAD patients could alter cerebrospinal fluid (CSF) flow given the lack of traditional pulsatile blood pressure [3]. The efficacy of CT myelograms has thus been questioned in these patients, increasing the level of diagnostic complexity in already inherently difficult cases. Furthermore, spinal pathology may additionally limit these patients' mobility, and any surgical intervention exposes these already at-risk patients to additional morbidity [4,5]. To our knowledge, we present the first case of an LVAD patient undergoing diagnostic evaluation via C1-C2 puncture for CT myelogram and subsequent cervical spinal surgery. We discuss the challenges of a diagnostic evaluation, surgical management, and mechanisms for these limitations.

## Case presentation

A 35-year-old male presented with the acute onset of neck pain and lower extremity weakness in the setting of hospitalization for sepsis with persistent leukocytosis and blood cultures positive for Streptococcus viridans and Candida glabrata. His past medical history included hypertension, type II diabetes mellitus, end-stage renal disease on dialysis, and non-ischemic cardiomyopathy. He had a history of a HeartMate 3 LVAD implantation four months prior complicated by redo-sternotomy for atrial valve replacement. He subsequently underwent tracheostomy and gastrostomy for airway and nutrition, respectively. At his baseline, he was able to ambulate independently, although he used a wheelchair in most instances. During his hospitalization, he also tested positive for COVID-19 (coronavirus disease 2019).

On physical exam, the patient had significant atrophy in his right lower extremity with flaccid paralysis and complete loss of tone. He had 2/5 strength in all muscle groups in the left lower extremity. He had a normal tone, was full strength, and normo-reflexive in the bilateral upper extremities. He was also found to be insensate in the right lower extremity and noted to have decreased sensation in the left lower extremity. His decline in the neurologic exam was partly attributed to critical illness polyneuropathy given his prolonged hospital and intensive care unit stay.

Diagnostic evaluation

**Figure 1:**
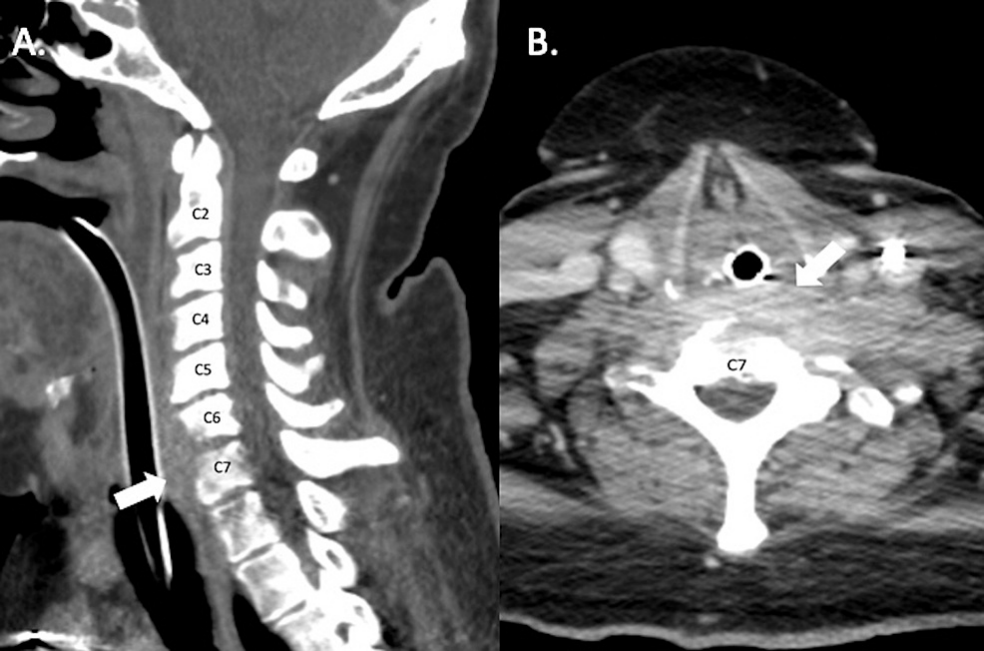
Initial Computed Tomography Scan of the Cervical Spine A. Sagittal view of computed tomography scan with intravenous contrast performed before obtaining computed tomography myelogram that demonstrates the erosion of the C6 and C7 vertebral bodies anteriorly with prevertebral phlegmon and a 7 mm prevertebral fluid collection concerning for abscess (white arrow). B. Axial view of C6-C7 level displaying the prevertebral collection (white arrow).

**Figure 2:**
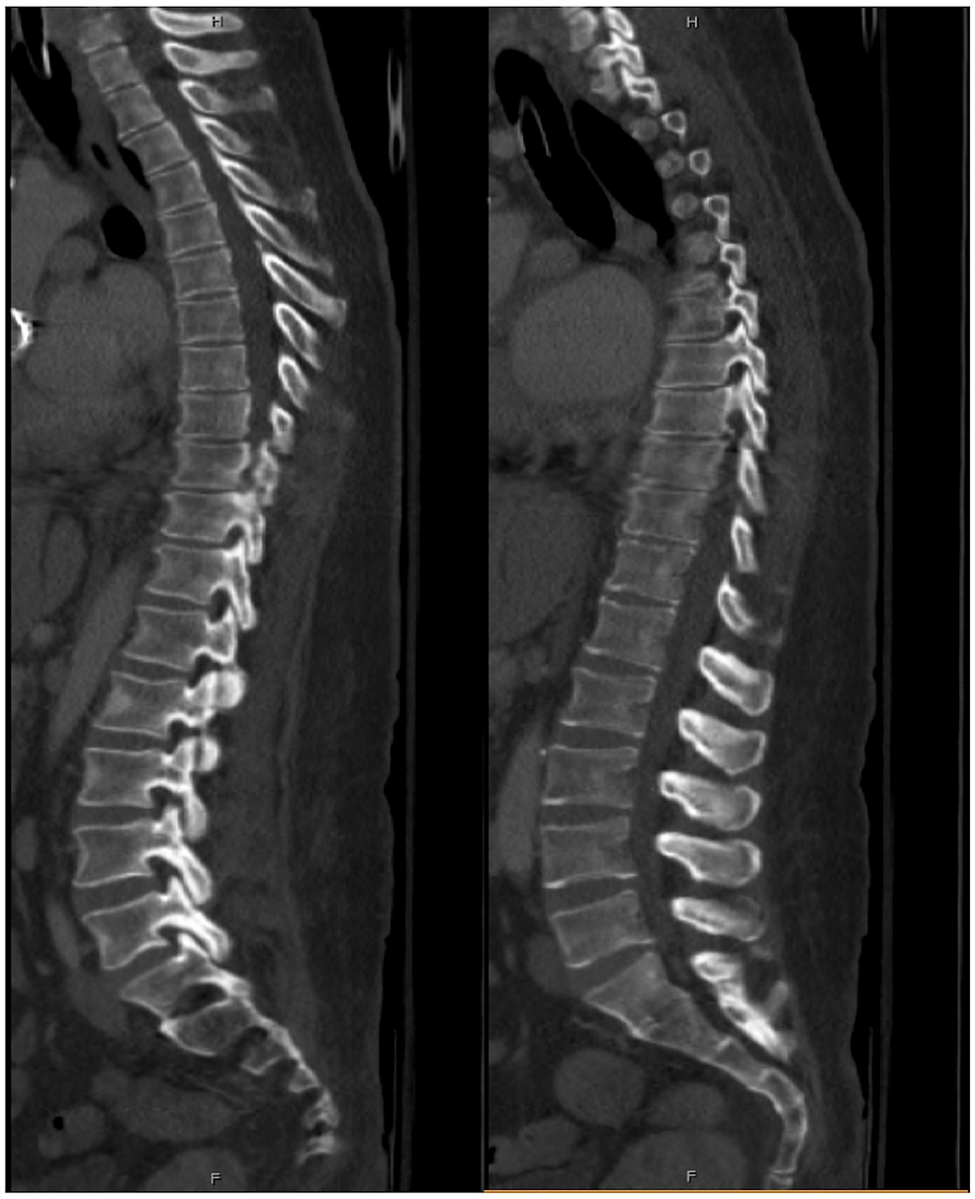
Initial Computed Tomography Scan of the Thoracic Spine Multiple sagittal views of the pre-operative computed tomography scan of the thoracic spine were found to be completely unremarkable.

A CT scan with contrast was initially performed that demonstrated mild erosion of the C6 and C7 vertebral bodies anteriorly with prevertebral phlegmon and a 7 mm prevertebral fluid collection concerning abscess, although there was no epidural collection or evidence of cord compression (Figure 1). A CT scan of the thoracic spine was also performed, which was completely unremarkable (Figure 2). Due to the patient’s LVAD, a CT myelogram was then performed instead of MRI. A cervical myelogram at C1-C2 was performed given the location of the suspected pathology, concern for dilution of the contrast media if conducted via lumbar puncture, and the need to visualize the superior border of the presumed pathology for pre-operative planning. This approach was chosen over lumbar injection due to concern that dye injected in the lumbar region would not travel to the region of interest. A 20-gauge, 10 cm needle was introduced into the cervical subarachnoid space at the level of C1-C2 under fluoroscopy, and 15 mL of clear CSF was obtained. 10 mL of iohexol-300 contrast was subsequently injected and the myelogram was performed. There were no complications, and the patient then underwent a CT scan of the cervical spine.

**Figure 3:**
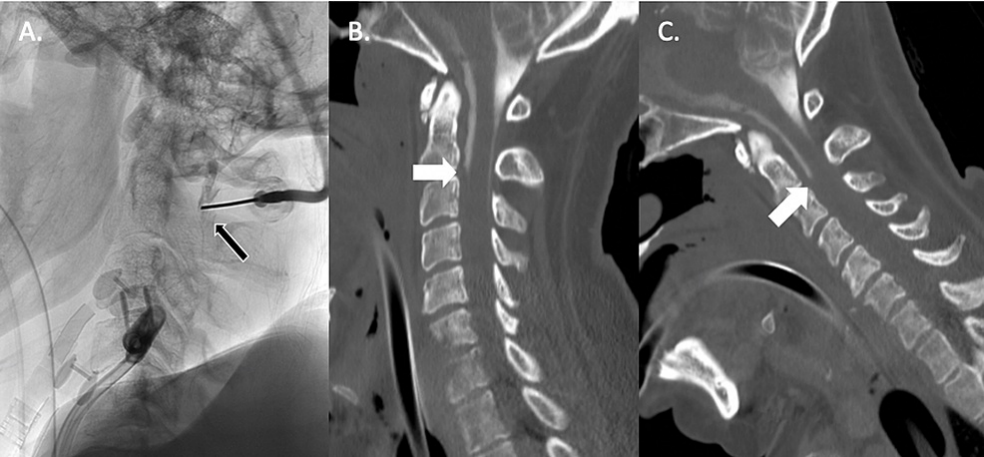
Cervical Computed Tomography Myelogram A. Lateral x-ray of the cervical spine during myelogram procedure. Percutaneous injection of iohexol 300 contrast via a 20-gauge needle at the C1-C2 level (black arrow). B. Sagittal view of computed tomography myelogram after C1-C2 lateral injection of contrast that demonstrates essentially no flow of contrast inferiorly past the level of C3. There is minimal movement down the dorsal and lateral epidural spaces at the level of C2-C3 (white arrow). Changes in discitis and osteomyelitis are again seen at C6-C7 anteriorly. C. Sagittal view with the neck in flexion to facilitate movement of contrast in the subarachnoid space. There is still no flow past the level of C3 (white arrow).

The CT myelogram imaging demonstrated a near-complete block of the ventral epidural space at the C2-C3 level with no to minimal contrast moving down the dorsal and lateral epidural spaces (Figure 3). Changes of discitis and osteomyelitis were again seen at C6-C7 anteriorly and on the left side of the pre-vertebral space.

Surgery

After discussion with the infectious disease, neurology, neurosurgery, cardiac surgery, cardiac anesthesia, and cardiac ICU teams, a circumferential epidural compressive pathology was felt to be the reason for the patient’s symptomology. This was based on the symptomology and fluid collection and phlegmon seen on the CT of the cervical spine, as well as lack of contrast advancement on the CT myelogram. While other diagnoses were considered that would not improve with surgical intervention, based on the patient’s young age and potential to benefit based on a compressive etiology, surgery was recommended.

An emergent C3-C7 posterior cervical laminectomy at the level seen on the CT myelogram was planned with the use of intra-operative ultrasound. During the procedure, no dorsal or ventral epidural purulence or signs of infection was noted via gross visualization and ultrasonic probing. There were no other compressive lesions at this location.

After decompression, the spinal cord appeared to be well decompressed circumferentially on ultrasound with surrounding CSF. The lamina and spinous processes were sent for permanent specimens, and a culture was performed of the epidural space. There were no intra-operative complications.

Postoperative course

**Figure 4:**
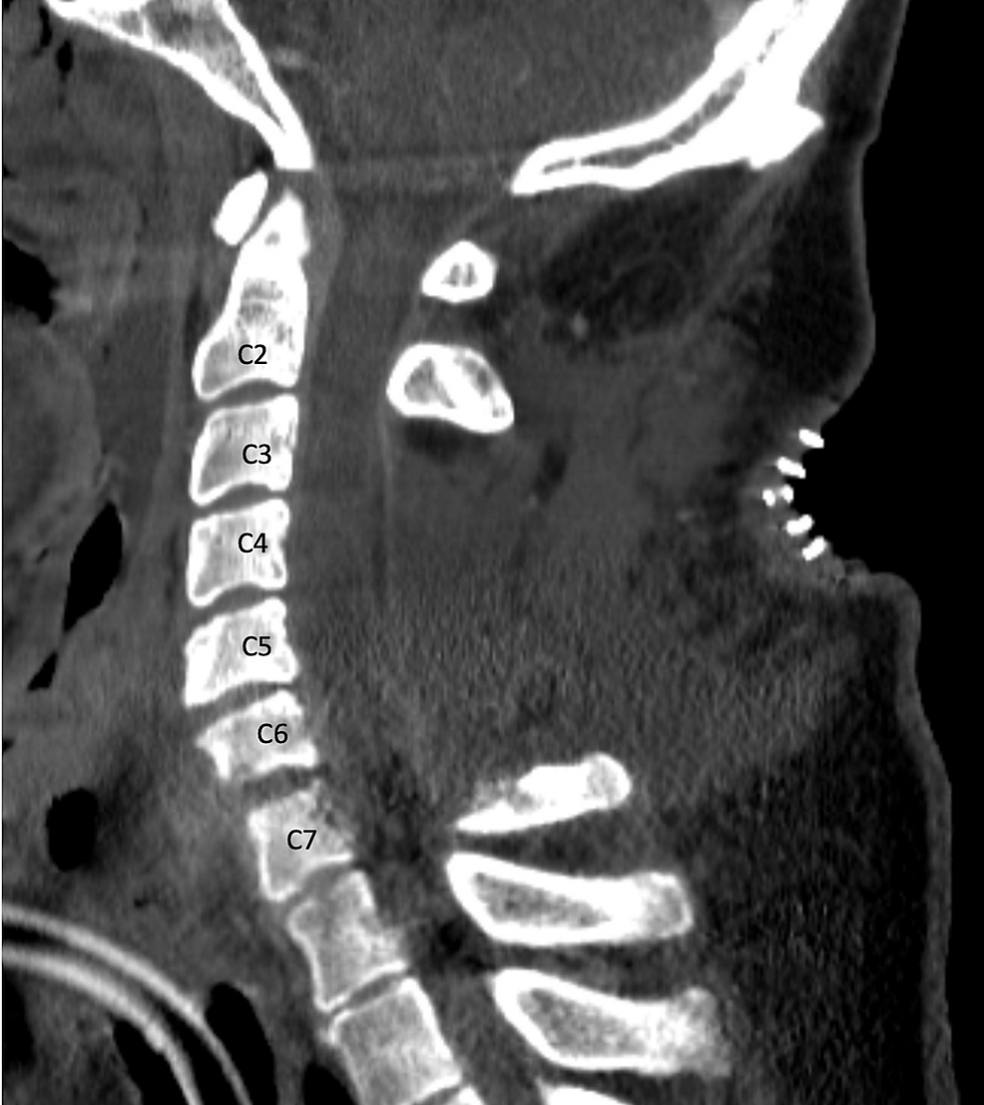
Postoperative Computed Tomography Scan Sagittal view of CT with contrast demonstrating postoperative changes after C3-C7 posterior laminectomy.

Postoperatively and at three-month follow-up, the patient remained neurologically stable with bilateral lower extremity paraparesis and minimal sensation. The cultures taken during the operation were negative, and the permanent specimens were found to be unremarkable for signs of infection. This result could, however, be confounded as the patient had previously received empiric antibiotics. Additional CT imaging demonstrated post-operative changes without additional pathology (Figure 4). Due to his lack of functional recovery and intra-operative findings and based on the clinical exam he was diagnosed with a suspected spinal cord infarct.

## Discussion

To our knowledge, this is the first reported case of a patient with an LVAD undergoing cervical spine evaluation with a cervical CT myelogram with subsequent posterior cervical spine surgery. A previous report by Kollmar et al. described a patient undergoing CT myelogram via lumbar puncture contrast injection and subsequent posterior lumbar decompression and fusion due to lumbar stenosis [6]. Our case uniquely involves the use of a contrast injection via a C1-C2 lateral approach, which is rarely used given advanced imaging techniques and potential complications [7]. This approach was specifically chosen based on the location of the patient’s pathology, symptomology, and concern that contrast dye injected in the lumbar spine would not travel sufficiently to the region of interest due to altered CSF based on LVAD physiology as well as the suspected location of spinal pathology.

In this case, a compressive etiology for the patient’s symptoms and exam findings was suspected due to lack of contrast extravasation past C2-3 as well as infectious changes seen at the level of C6 on CT scan. However, the intra-operative surgical evaluation did not seem to reveal a compressive etiology. Therefore, a spinal cord infarct was suspected. This highlights the complexity involved in the evaluation of these patients, and unfortunately, there is little to no current literature available to help guide diagnosis.

It is not entirely clear what prevented contrast from extending down the spinal column, although there are possible explanations. Recent work by Mestre et al. has demonstrated that CSF flow is dependent on arterial pulsations and driven by the cardiac cycle [8]. Interestingly, current LVAD models do not rely solely on pulsatile fluid dynamics and instead primarily on a continuous flow of blood that results in diminished blood pressure and reduction in pulse pressure [9]. Recent technological advancements in the HeartMate 3 have resulted in a mechanism that intermittently reduces and advances the continuous pump speed mimicking a pulse of 30 beats per minute [10]. This, however, may still not be able to mimic physiologic parameters that produce a bulk flow of CSF in a normal setting.

The results of this hypothesis remain unclear as CSF flow models in LVAD patients have not been successful to date [3]. One study using a computational model demonstrated that continuous flow resulted in a reduction in CSF pressure amplitude and velocity though the overall flow rate was unchanged [4]. It seems reasonable that the alteration of pulsatility contributed to an interruption of the advancement of contrast down the subarachnoid column. However, because the natural flow of spinal fluid alone is not solely responsible for contrast media movement during CT myelogram, this is likely not the only reason for the lack of contrast advancement. As patient body position is manipulated during CT myelogram, gravity also plays a significant role in contrast advancement. Thus, another possibility remains an underlying compressive pathology that simply was not grossly visible during the operation. Further, the potential remains for intrathecal inflammatory conditions such as adhesive arachnoiditis that can cause progressive paraplegia and prevent contrast advancement due to intrathecal scarring. As LVAD implantation rates increase, however, understanding the consequences relating to central nervous system physiology becomes important. Appropriately diagnosing compressive etiologies in the setting of progressive neurologic worsening is challenging but vital for the treatment of these patients.

Over the past several decades, significant efforts have been undertaken regarding the creation of MRI-compatible cardiac devices. Current MRI-compatible cardiac devices include pacemakers, ventricular partitioning devices, ventricular elastic support devices, and heart valves [2]. To our knowledge, there are no MRI-compatible LVADs in use and none currently undergoing human trials. As the number of patients with LVADs increases, and as the lifespan of these patients continues to increase with technological advancements, the prevalence of LVAD patients benefiting from MRI imaging will subsequently increase. Future research is therefore warranted regarding MRI-compatible LVADs.

Previous non-cardiac surgeries and the reported case of lumbar spine surgery in a patient with an LVAD have all shown the potential for intraoperative complications related to prone positioning, coagulopathies, and alterations in LVAD flow dynamics [6,11]. In the case of our patient, anticoagulation was held for three days as the patient had recently been extubated and experienced issues with tracheal and oropharyngeal bleeding. Estimated blood loss was relatively low for the operation (estimated at 125 mL). It is important to note that even with a normal INR, however, these patients remain coagulopathic as nearly 100% of them experience an acquired Von Willebrand disease due to LVAD flow physiology [12].

In regards to prone positioning for spinal surgery, specific attention must be given to the placement of the LVAD driveline and the location of the outflow cannula of the aorta [6]. Further, prone positioning has been noted to decrease preload to the left ventricle, which can be especially disruptive in a patient with an LVAD as the device operates on specific inflow and outflow parameters [4,6,13]. This has been reported to lead to intra-operative hypotension [6]. The presence of an experienced cardiac anesthesia team, careful positioning pre-operatively, and continual LVAD monitoring minimize risks related to these factors.

## Conclusions

As the number of patients with LVADs increases, the frequency of patients requiring spinal imaging and subsequent surgery will rise. Unfortunately, these patients have inherently high surgical risk and remain complex to evaluate diagnostically. Currently, CT myelogram remains the diagnostic imaging of choice for patients with LVADs who are unable to undergo MRI and require visualization of the spinal neural elements. Further research is warranted as current experience is limited, and it is unclear if this modality is in any way distorted by altered CSF physiology. If posterior spinal surgery is warranted in these patients, it can be safely performed with a multi-disciplinary approach and careful attention to patient positioning.

## References

[1] Yancy CW, Jessup M, Bozkurt B (2017). 2017 ACC/AHA/HFSA focused update of the 2013 ACCF/AHA guideline for the management of heart failure: a report of the American college of cardiology/American heart association task force on clinical practice guidelines and the heart failure society of America. Circulation.

[2] Sigakis CJ, Mathai SK, Suby-Long TD (2018). Radiographic review of current therapeutic and monitoring devices in the chest. Radiographics.

[3] Luc JG, Pierre CA, Phan K (2017). Fluid structure interaction model analysis of cerebrospinal fluid circulation in patients with continuous-flow left ventricular assist devices. Int J Artif Organs.

[4] Edgcombe H, Carter K, Yarrow S (2008). Anaesthesia in the prone position. Br J Anaesth.

[5] Degnan M, Brodt J, Rodriguez-Blanco Y (2016). Perioperative management of patients with left ventricular assist devices undergoing noncardiac surgery. Ann Card Anaesth.

[6] Kollmar JP, Colquhoun DA, Huffmyer JL (2017). Anesthetic challenges for posterior spine surgery in a patient with left ventricular assist device: a case report. A A Case Rep.

[7] Yousem DM, Gujar SK (2009). Are C1-2 punctures for routine cervical myelography below the standard of care?. AJNR Am J Neuroradiol.

[8] Mestre H, Tithof J, Du T (2018). Flow of cerebrospinal fluid is driven by arterial pulsations and is reduced in hypertension. Nat Commun.

[9] Lalonde SD, Alba AC, Rigobon A (2013). Clinical differences between continuous flow ventricular assist devices: a comparison between HeartMate II and HeartWare HVAD. J Card Surg.

[10] Chatterjee A, Feldmann C, Hanke JS (2018). The momentum of HeartMate 3: a novel active magnetically levitated centrifugal left ventricular assist device (LVAD). J Thorac Dis.

[11] Roberts SM, Hovord DG, Kodavatiganti R, Sathishkumar S (2015). Ventricular assist devices and non-cardiac surgery. BMC Anesthesiol.

[12] Dassanayaka S, Slaughter MS, Bartoli CR (2013). Mechanistic pathway(s) of acquired von willebrand syndrome with a continuous-flow ventricular assist device: in vitro findings. ASAIO J.

[13] Slaughter MS, Pagani FD, Rogers JG (2010). Clinical management of continuous-flow left ventricular assist devices in advanced heart failure. J Heart Lung Transplant.

